# Chemical changes in organic matter after fungal colonization in a nitrogen fertilized and unfertilized Norway spruce forest

**DOI:** 10.1007/s11104-017-3324-8

**Published:** 2017-07-08

**Authors:** César Nicolás, Juan P. Almeida, Magnus Ellström, Adam Bahr, Sharon E. Bone, Nicholas P. Rosenstock, John R. Bargar, Anders Tunlid, Per Persson, Håkan Wallander

**Affiliations:** 1 Microbial Ecology Group, Department of Biology, Lund University, Lund, Sweden; 2 Department of Biology, Ecology Building, Lund University, SE 22362 Lund, Sweden; 3 Chemistry and Catalysis Division, Stanford Synchrotron Radiation Lightsource, SLAC National Accelerator Laboratory, Menlo Park, CA USA; 4 Centre for Environmental and Climate Research, Lund University, Lund, Sweden

**Keywords:** Fungal community composition, Infrared spectroscopy, Near-edge X-ray absorption fine structure (NEXAFS) spectroscopy, Nitrogen fertilization, Norway spruce forest, Organic matter decomposition

## Abstract

**Background and aims:**

Decomposition and transformation of organic matter (OM) in forest soils are conducted by the concomitant action of saprotrophic and mycorrhizal fungi. Here, we examine chemical changes in OM after fungal colonization in nitrogen fertilized and unfertilized soils from a Norway spruce forest.

**Methods:**

Sand-filled bags amended with composted maize leaves were placed in the forest soil and harvested after 17 months. Infrared and near edge X-ray absorption fine structure spectroscopies were used to study the chemical changes in the OM. Fungal community composition of the bags was also evaluated.

**Results:**

The proportion of ectomycorrhizal fungi declined in the fertilized plots, but the overall fungal community composition was similar between N treatments. Decomposition of the OM was, independently of the N level or soil horizon, accompanied by an increase of C/N ratio of the mesh-bag content. Moreover, the proportions of carboxylic compounds in the incubated OM increased in the mineral horizon, while heterocyclic-N compounds decreased, especially in unfertilized plots with higher N demand from the trees.

**Conclusions:**

Our results indicate that more oxidized organic C and less heterocyclic-N proportions in the OM remain after fungal colonization in the mineral layers, and suggest that ectomycorrhizal fungi transfer less heterocyclic-N from the mesh bags to the host trees under high N levels.

**Electronic supplementary material:**

The online version of this article (doi:10.1007/s11104-017-3324-8) contains supplementary material, which is available to authorized users.

## Introduction

Saprotrophic fungi are one of the major decomposers of organic matter (OM) in boreal and northern temperate forests. These microorganisms harbour a wide range of hydrolytic and oxidative enzymes such as cellobiohydrolases, laccases, peroxidases or lytic polysaccharide mono-oxygenases that allow them to break down plant litter (Baldrian 2006; Bugg et al. 2011; Hemsworth et al. 2013; Kohler et al. 2015) and use it as a C source. In addition to saprotrophic fungi, ectomycorrhizal (ECM) fungi constitute another major microbial group in boreal and northern temperate forests and may play an important role in OM decomposition. Unlike saprotrophs, ECM fungi forage the SOM to obtain nutrients such as N and P for the host plant and receive in return plant photosynthate C. Nevertheless, the ability of ECM fungi to produce oxidative enzymes suggests a potential role for these microbes in the decomposition of soil OM (Bödeker et al. 2014; Phillips et al. 2014). Furthermore, recent studies have shown that ECM fungi have retained similar oxidative capacity as saprotrophic fungi (Shah et al. 2016). This oxidative capacity has been suggested to primarily assist the ECM fungi to mobilizing nutrients from soil OM such as N embedded in the soil organic complexes, rather than to obtain C from the soil OM (Lindahl and Tunlid 2015; Shah et al. 2016). However, decomposition and chemical changes of OM after ECM colonization are still poorly understood.

Fungal community composition is altered by N fertilization (Morrison et al. 2016). Nitrophylic fungal species more adapted to high N levels can proliferate in N fertilized soils and the diversity of saprotrophs, such as ascomycetes, which are more capable of degrading cellulose, is enhanced under N fertilization (Morrison et al. 2016). In addition, N fertilization reduces fungal biomass (Frey et al. 2014; Bahr et al. 2015), and lowers oxidase and hydrolytic activities (Allison et al. 2008; Rinkes et al. 2016). These changes in fungal composition and activity may be reflected in the chemical composition of OM. For instance, a lower fungal biomass can result into a reduced contribution of the fungal derived products (e.g. chitin and melanin from necromass, secreted enzymes) to the soil OM. Likewise, lower oxidative and proteolytic activity of saprotrophic and ECM fungi when easily available N such as ammonium forms are present, may reduce the decomposition of lignocellulosic and organic N compounds. Despite the important role of fungi in decomposition of OM in forest soils, there is still a lack of knowledge regarding how fungal colonization will affect the chemical composition of OM.

Spectroscopic methods are useful tools to study the composition of soil OM (Kögel-Knabner 2000; Lehmann et al. 2009). In this study, we combine the use of infrared spectroscopy and X-ray absorption spectroscopy to characterize the changes in the soil OM taking place after fungal colonization of OM added to fertilized and unfertilized forest plots. Infrared spectroscopy yields information about the chemical groups in soil OM and provides a fingerprint of the composition of the OM (Bouskill et al. 2016). Synchrotron-based near-edge X-ray absorption fine structure (NEXAFS) spectroscopy is used to determine the chemical environment of C and N in the soil samples (Solomon et al. 2012; Leinweber et al. 2013; Gillespie et al. 2015). In comparison with other techniques such as infrared or nuclear magnetic resonance (NMR) spectroscopies, NEXAFS spectroscopy is very sensitive to element speciation and has been applied to soil samples in order to elucidate chemical changes in C and N of soil OM due to effects of land management (Solomon et al. 2005), N fertilization (Gillespie et al. 2013) or climate change (Purton et al. 2015).

The aim of this study was to examine changes in the chemical composition of OM after fungal colonization in fertilized and unfertilized forest soils. OM in the form of maize compost was added to sand-filled mesh bags that prevented in-growth of roots. Since maize is a C4 plant, its use as organic material allowed for quantifying the amount of new carbon (C3-carbon) that entered the mesh bags during incubation in the soil (Wallander et al. 2011). The mesh bags were placed along the soil profile from the organic to the mineral horizon to capture fungal communities from these horizons (Lindahl et al. 2007). Fungal colonization and community composition in the mesh bags were assessed at the end of the soil incubation period. The first hypothesis of the study was that there would be an increase of the C/N ratio of the organic material of the mesh bags due to a preferential removal of N by the ECM fungi. Furthermore, we expected this effect to be larger in the unfertilized plots where the demand for N is higher. Our second hypothesis was that the proportion of organic N pools would change during incubation since ECM fungi were expected to transfer some organic N pools to the plant host, while other pools would accumulate as residues of fungal necromass.

## Materials and methods

### Site and experimental design

The experiment was conducted at Tönnersjöheden research park (56°41′N, 13°5′E) in the county of Halland, Southwestern Sweden. The annual precipitation is 800–1050 mm and the average temperature ranges between 6.1–7.3 °C. The soil type is a Haplic Podzol according to IUSS Working Group WRB (2006), and its texture is sandy. The experimental site was afforested with Norway spruce (*Picea abies*) and established in 1979.

A total of six plots (30–40 m by 25 m) were selected for the experiment: three of these were amended with 200 kg N ha^−1^ in the form of NH_4_NO_3_ in July 2011, and three were used as control. At the same time, cylindrical mesh bags (length 16 cm and diameter 2 cm) filled with 70 g acid-washed quartz sand (0.36–2.00 mm, 99.6% SiO_2_, Ahlsell AB, Malmö, Sweden) and 2% dry weight composted maize materials were placed in the experimental plots. The compost material was produced by composting chopped maize leaves for 12 months in an isolated compost bin placed outside. After composting, the material was stored at 4 °C until use in the experiment. The use of composted maize leaves was selected as organic material in order to estimate the amount of fungal C entering the mesh bags from the surrounding soil. This was done by analyzing the change in the δ^13^C in the compost material originated from maize leaves (C4 plant) when colonized by fungi from the soil (C originated from C3 plants) (Wallander et al. 2011). A total of six mesh bags per plot were vertically installed along the soil profile from the humus horizon to the mineral horizon. The depth of the humus horizon at each site was recorded. The mesh size of the bags (50 μm) allowed fungal hyphae to penetrate the bags but excluded the roots, and enabled the estimation of the accumulation of ectomycorrhizal biomass in the mesh bags (Wallander et al. 2001). The bags were collected in November 2012, and the contents of the bags were divided into two groups. One corresponded to the humus horizon at the site where the bags were placed, and the other corresponded to the upper mineral soil. The bags were subsequently stored at −20 °C until chemical or biological analysis. The contents of all mesh bags from a given soil horizon in a single plot were pooled and mixed to obtain one sample for each soil horizon per plot.

### Analysis of the fungal colonization and community composition in the mesh bags

Fungal colonization in the mesh bags was estimated using three different methods: scoring, counting and ergosterol measurement. The scoring method consisted of visual estimation of the extramatrical mycelium with a stereomicroscope using a 6-graded scale (Wallander et al. 2001). The counting method examined the frequency of hyphae present in mesh-bag holes along vertical rows in increments of 100 mesh holes. The ergosterol method consisted of the extraction and quantification of the fungal-specific membrane lipid ergosterol via high-performance liquid chromatography coupled with a UV detector (Bahr et al. 2015).

The fungal community composition in mesh bags and the initial maize material was characterized after DNA extraction, amplification, sequencing and analysis of the fungal DNA. The DNA was extracted from 5 g of homogenized ground material from each composite sample obtained from six mesh bags with a CTAB buffer (2% cetyltrimethylammonium bromide, 2 mM EDTA, and 150 mMTris-HCl, pH 8) and incubated at 65 °C for 1.5 h, followed by chloroform centrifugation and isopropanol/ethanol precipitation, and purification with Nucleo-spin DNA clean up kit (Machery-Nagel, Düren, Germany). Three technical replicates were performed for each sample for extraction, PCR, and sequencing.. Negative controls were used for the extraction and amplification steps and consisted of 3 ml MilliQ water instead of 5 g mesh bag contents.

The ITS2 region was amplified using fungal-specific primers ITS7g (Ihrmark et al. 2012) and ITS4 (Gardes and Bruns 1993). The ITS2 region and these primers exhibit low bias for high-throughput sequencing studies due to lower length variation of the amplicon and reduced primer bias (Ihrmark et al. 2012). The forward and reverse primers contained adapters used to attach the barcodes in the next steps. Each 25 μl of PCR reaction consisted of 0.25 μl Phusion polymerase® (Thermo Scientific, Waltham, MA, USA), 5 μl Buffer 5X, 0.5 μl dNTPs (10 mM), 1.5 μl ITS7g (10 μM), 1.5 μl ITS4 (10 μM), 1 μl BSA (20 mg/ml), and 1.5 μl of template DNA (5 ng/μl). After PCR (initial denaturation at 98 °C for 30 s; then 31 cycles of 98 °C for 10 s, 56°Cfor 30 s, and 72 °C for 30 s, with a final extension for 8 min at 72 °C), the products were subjected to AMPure® purification (AgencourtAMPure XP, Beckman Coulter, Brea, CA, USA) to remove primers, dNTPs, short fragments, and sequencing inhibitors. A second 8-cycle amplification was then performed to attach Nextera sample-specific barcodes (Illumina Inc., San Diego, CA, USA) for sorting after sequencing. Following the Nextera amplification, PCR products were subjected to an additional AMPure® purification, and the purified PCR products were measured using a Quant-iT™ PicoGreen® dsDNA Assay Kit (Invitrogen™, Carlsbad, CA, USA). Equal amounts of DNA from each sample were then pooled and submitted for Illumina sequencing with paired end (325 bp forward; 275 bp reverse) sequencing on MiSeqsequenator using the MiSeq Reagent Kit v3 chemistry (Illumina Inc., San Diego, CA, USA). Negative extraction and PCR controls yielded no detectable DNA. All sequences were submitted to the International Nucleotide Sequence Database Sequence Read Archive (accession no. SRR3591717).

The sequences were trimmed and filtered using Mothur v1.34 (Schloss et al. 2009). Sequences outside the ITS2 region and chimeric sequences were removed using ITSx extractor v1.5.0 (Bengtsson-Palme et al. 2013). After filtering, the sequences were clustered using the Gaussian Mixture model CROP (Hao et al. 2011) at 97% sequence similarity, and a set of operational taxonomic units (OTUs) were thus obtained. The taxonomic identity was then assigned to the set of clustered sequences by searching the Full “UNITE + INSD” dataset (Kõljalg et al. 2013) using the Basic Local Alignment Tool (BLASTN program 2.2.25,blast.ncbi.nlm.nih.gov). Sequences that presented a 96% similarity between the query sequence and top hit, with at least 80% coverage of the query sequence length, were assigned to a taxonomic identity with genus and species. Sequences with values of 94–95% similarity between the query sequence and top hit were considered to be assigned a taxonomic identity at the genus level only.

All OTUs which represented less than 10 total reads and occurring in only one sample were removed from the OTU abundance matrix and read abundances within each OTU were averaged between the technical replicates for each sample. Rarefaction was performed to the median number of reads across all samples, 17,345 reads. Read abundances for all OTUs for each sample were then summed and expressed as the relative abundance per sample, such that total abundance for each sample was 1. This rarefied relative abundance matrix containing the abundance of OTUs per sample was used to analyze the community composition differences. The total fungal community was divided by ecological function (ECM fungi, saprotrophic fungi, plant pathogens and unknown ECM fungi); OTUs were considered to be known ECM fungi based on the most current knowledge of the ecology of known close relatives (genera or species) according to Tedersoo et al. (2010). The assignment of the remaining OTUs was done to the best of our knowledge. For data visualization, only OTUs with greater than 50 reads were shown (representing >99% of the total reads).

### Chemical analysis of the organic matter in the mesh bags

Total organic C and total N contents of the samples from the mesh bags, as well as from the initial maize material that had not been incubated in the soil, were measured using a TOC analyzer equipped with a TNM-1 detector (Shimadzu). The pH of the incubated mesh bags was measured in a 1:5 dilution of mesh-bag content: water. Nitrate and ammonium of the initial mesh-bag content were determined by flow injection analysis (FIAstar 5000, Foss, Denmark) on an extract obtained after shaking the material for 2 h with a 2 M KCl solution (1:20, *w*/*v*).

In order to estimate the import of C to the mesh bags, the ^13^C in the samples was analyzed (Wallander et al. 2011). The determination of the natural abundance of ^13^C in the samples from the mesh bags and from the initial maize material was performed with an isotope ratio mass spectrometer (Isoprime, Manchester, UK). The isotopic composition of the samples was expressed as deviations relative to standard reference materials:

$$ {\delta}^{13} C\kern0.5em \left({\mbox{\fontencoding{U}\fontfamily{wasy}\selectfont\char104}} \right)=\left(\frac{R_{sample}-{R}_{standard}}{R_{standard}}\right)\ast 1000 $$

where R is the molar ratio of ^13^C/^12^C. The standard reference material for C was the Vienna Pee Dee Belemnite limestone. The proportion of new C in the mesh bags was calculated with a two component mixing model:

$$ {C}_4- carbon=\left(\frac{\delta^{13}{C}_{sample}-{\delta}^{13}{C}_{new\  carbon}}{\delta^{13}{C}_4-{\delta}^{13}{C}_{new\  carbon}}\right) $$

where the values of δ^13^C_new carbon_ were obtained from extracted mycelia from mesh bags with sand from a similar forest (−25.82‰ ± 0.02, *n* = 2), and the value δ^13^C_4_ was determined from the initial maize compost (−13.36‰ ± 0.02, *n* = 3).

Fourier Transform Infrared (FTIR) spectra were recorded using a Bruker Tensor 27 (Bruker Scientific Instruments, Billerica, MA, USA). The data were collected in diffuse reflectance mode on freeze-dried dissolved OM mixed with potassium bromide (2:100, m/m). The dissolved OM was obtained by extracting the mesh bag content using distilled water (1:5, m/v) for 24 h under agitation. The solution was then filtered through 0.22 μm and freeze-dried. Each spectrum was the result of 128 consecutive scans at a resolution of 4 cm^−1^. Six vibrational modes of the spectra were mainly used to identify changes of the functional groups in the OM: C-O and C-O-C stretching of carbohydrates (970–1150 cm^−1^) and C-O stretching of phenolics (1150–1250 cm^−1^), N-O stretching of nitrates or N-H bending of ammonium (1380–1400 cm^−1^), O-H bending and aliphatic C-H deformation (1350–1450 cm^−1^), C-C stretching of aromatic rings (1510 cm^−1^) and C = O stretching of carbonyl groups (1620–1800 cm^−1^).

NEXAFS spectra were collected at the beamline 11ID-1 (SGM) of the Canadian Light Source facilities in Saskatoon, Canada. The organic material from the mesh bags, as well as the initial maize material, was ground and slurried in water (1:160, *w*/*v*). An aliquot of 50 μl of the samples were deposited on 5 × 5 mm^2^ indium foil (Sigma Aldrich), which had previously been cleaned with acetone and ethanol, and the sample was allowed to dry on a hot plate at 30 °C in a laminar flow cabinet. This process was repeated six times. The indium foil was fixed to NEXAFS sample holders using double-sided carbon tape. The measurements were performed in fluorescence mode and each scan was collected from fresh, unexposed locations on the sample. The spectra were recorded using a slew scanning mode at the C K-edge (275–320 eV) and the N K-edge (390–430 eV) in order to minimize the X-ray exposure, and a minimum of 8 scans were collected per sample. The beam spot size was 1000 × 100 μm^2^ and the beamline exit slit was 25. The energy resolution of the measurement (E/ΔE) was higher than 5000. For calibrations of the C and N K-edges, the absorption spectra for citric acid and ammonium sulfate were measured, respectively. Normalization of N K-edge spectra was done by using the beamline flux (I_0_) using an in-line gold mesh, while that of C K-edge spectra was done by measuring the I_0_ on a freshly gold-sputtered film located at the sample chamber. Additionally, all spectra were normalized to an edge jump equal to 1.

Spectral deconvolution was done by using Gaussian curves and error functions with the program ‘fityk –version 0.9.8’ (Wojdyr 2010) (Fig. S1). Initially, error functions were fitted to the edge jumps and then a series of Gaussian curves were centered according to the energy position of C and N species found in literature. Thus, Gaussian curves were set at 284.3, 285.3, 286.0, 286.6, 287.4, 288.4 and 289.3 eV in the NEXAFS C1s spectra corresponding to quinone-C, substituted aromatic-C, aromatic-C/pyridinic-C, phenolic-C, aliphatic-C, carboxyl-C and O-alkyl-C, respectively (Solomon et al. 2009; Gillespie et al. 2014; Purton et al. 2015). Gaussian curves for the N K-edge were placed at 398.8, 400, 401.4, 402.7 and 405.4 eV corresponding to heterocyclic-N (e.g. pyrimidine, pyridine), nitrilic/heterocylic-N, amidic-N, pyrrolic-N and nitrate-N/ammonium-N, respectively (Leinweber et al. 2007, 2010). In addition, Gaussian curves before the edge were set to equal a full width at the maximum height of 1.2 eV, while those after the edge were not constrained before iteration. The relative abundances of the π* transitions were finally determined with respect to the sum of all π* transitions and were used as indicators of the content of major C and N types in the samples.

### Statistical analysis

All statistical analysis regarding fungal community composition was conducted using the VEGAN R package (Oksanen et al. 2016). Differences in fungal community composition between samples were calculated with Bray-Curtis dissimilarity and plotted in non-metric multidimensional scaling ordination plots (NMDS). The statistical significance of the differences in community composition was tested using permutation multivariate analysis of variances (PERMANOVA).

One-way ANOVA was used for the statistical analysis of fungal biomass and chemical parameters between, and a post-hoc Tukey’s honestly significant difference (HSD) test, was applied to establish differences between treatments. The Welch and Brown-Forsythe tests were applied to establish differences between groups in case of unequal variances. A two-tailed test of population mean with unknown variances was used when N types obtained from NEXAFS spectroscopy were compared with the initial material. The statistical comparison between horizons was not conducted, because both samples were not strictly independent. Principal component analysis (PCA) was performed on both the relative abundance of the peak area obtained from NEXAFS spectroscopy and the infrared spectral data. Infrared spectral data had previously been normalized to the same total area before PCA analysis. SPSS 13.0 software (IBM, USA) and R version 3.1.2 (R Development Core Team 2014) were used for data analysis.

## Results

### Fungal colonization and community in the mesh bags in the organic and mineral horizon

**Fig. 1:**
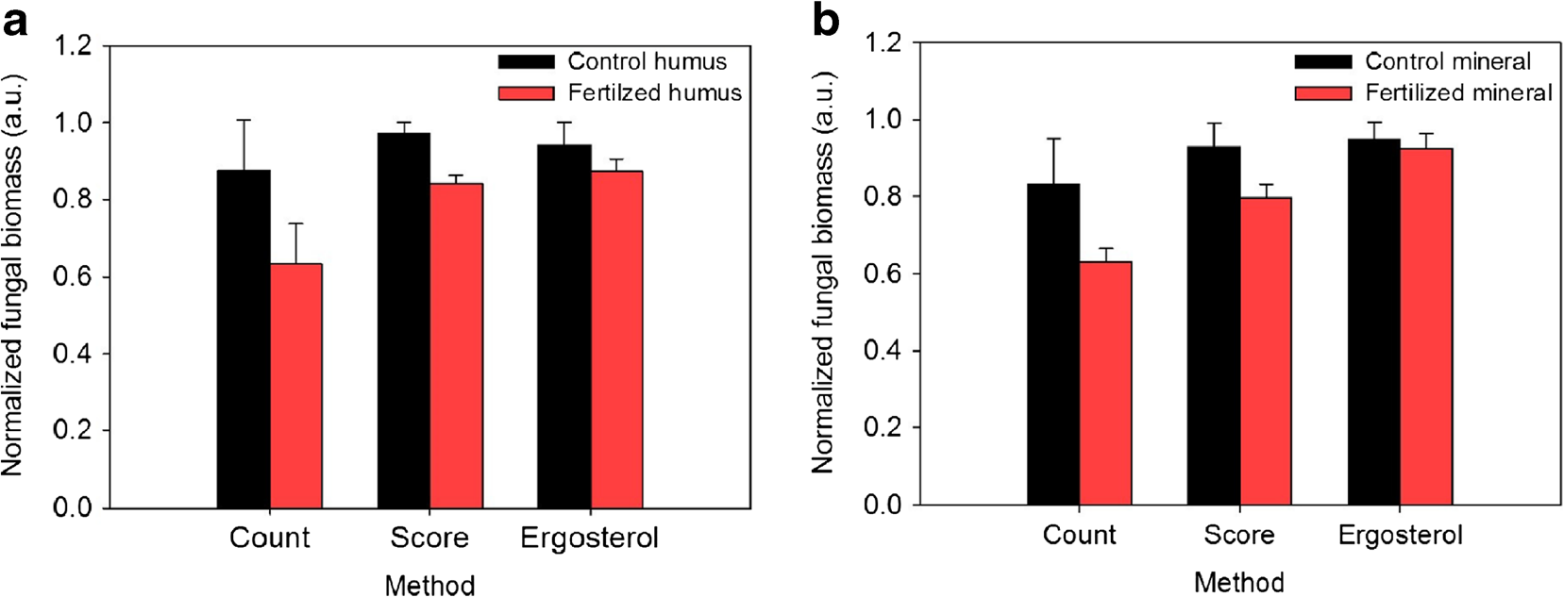
Normalized fungal colonization obtained from the mesh bags of the control (blue bars) and fertilized plots (red bars) after 17 months of incubation (**a**) in the organic and (**b**) in the mineral horizon determined by counting, scoring and ergosterol methods (error bars are standard errors, *n* = 3). Values are normalized to the maximum value of each method

Fungal colonization measured using the different methods (ergosterol, counts and score) tended to be lower in the mesh bags placed in fertilized plots at both soil horizons, although there were no significant differences among treatments (Fig. 1a, b).

**Fig. 2:**
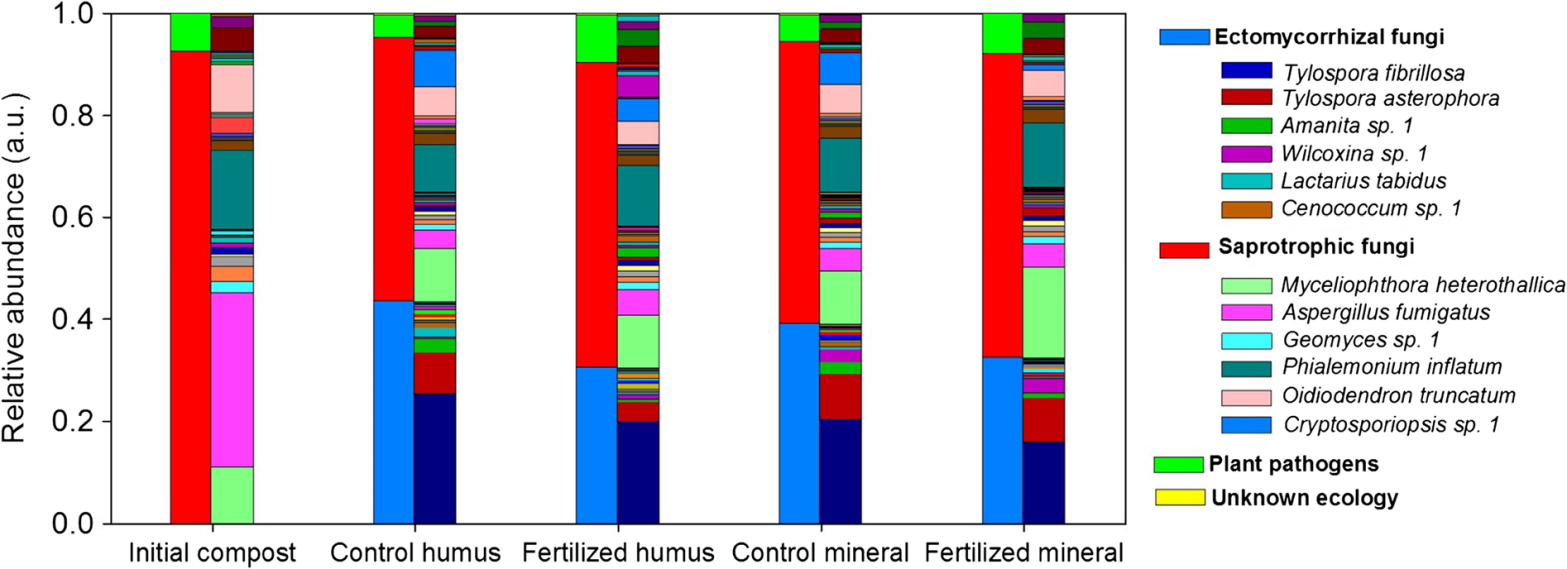
Relative abundance of fungal OTUs obtained from the mesh bags of the control and fertilized plots at the beginning of the experiment (initial material) and after 17 months of incubation in the organic and in the mineral horizon. The relative abundance was normalized to the number of reads. For each sample, the left stacked bar shows the fungal OTUs grouped according to their ecological role, and the right stacked bar shows all fungal OTUs, where a selected number of the most abundant OTUs (12 out of 83 OTUs) are indicated in the legend. Each bar represent the mean abundance of three replicates, except for the initial material that had one replicate. Only OTUs with greater than 50 sequence reads (representing 99% of the total sequence reads) are depicted. The list of ectomycorrhizal and saprotrophic fungi are shown in Fig. S3-S4

**Fig. 3:**
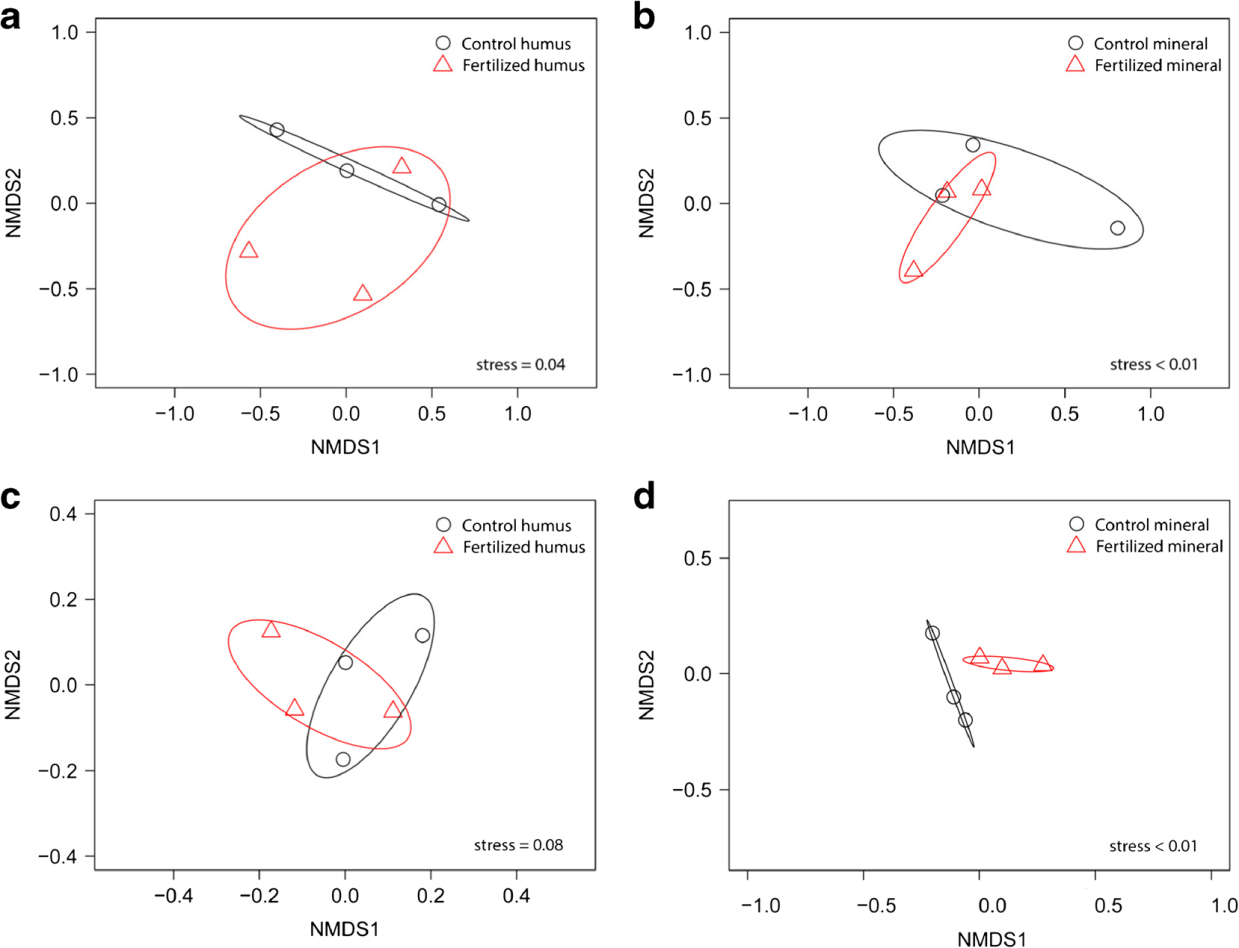
Non-metric multidimensional scaling (NMDS) ordination of ectomycorrhizal fungal OTUs (**a**, **b**) and all fungal OTUs (**c**, **d**) obtained from the mesh bags of the control and fertilized plots placed in the organic and in the mineral horizon (*n* = 3). Ellipses represent a 95% confidence interval around the mean for the different treatments

We obtained 814,942 sequence reads from sequencing and had 721,849 reads after quality filtering. Initial clustering resulted in 2257 OTUs. After taxonomic assignment, eliminating rare taxa (<10 reads) and taxa that only occurred in one sample and averaging of technical replicates we had 298 OTUs corresponding to 255,252 reads with a range of 13,810 to 24,843 reads per sample (Fig. S2a). Approximately 4% of the reads could not be assigned a taxonomic identity. Of those for which a taxonomic identity could be assigned, ecological role was inferred to 99.6% of the OTUs, which accounted for ECM fungi, saprotrophs and plants pathogens (Fig. 2). Compared to the initial compost material, the material incubated in the mesh bags at the organic and mineral horizons contained a significant proportion of ECM, which ranged from 30.6–43.5%. N fertilization was associated with a 10% lower ECM fungal abundance in the mesh bags at the humus and mineral horizons relative to the control (*p* < 0.05 and *p* = 0.06, respectively) (Fig. 2). In total, 59 ECM fungal species were identified (26 species if the abundance cut-off was more than 50 reads), with the most abundant being similar in the fertilized and control plots (Fig. 2, Fig. S2b, Fig. S3). The ECM fungi included *Tylospora fibrillosa* and *Tylospora asterophora,* which represented 67–85% of the total ECM species, followed by *Amanita* sp., *Wilcoxina* sp. and *Lactarius tabidus.* Nitrogen fertilization did not produce a significant change in ECM community composition as revealed by PERMANOVA analysis for the organic horizon (R^2^ = 0.18, *p* = 0.9) or the mineral horizon (R^2^ = 0.18, *p* = 0.6) (Fig. 3a, b).

Regarding the saprotrophic fungi, their fungal abundance ranged from 49.6–63.8% with respect to the total fungal abundance. A number of fungal species that were not present in the initial material (maize compost), proliferated in the mesh bags under field conditions and represented an average of 12% of the total fungal abundance. This was the case for saprotrophic *Cryptosporiopsis* sp., which was not present in the initial material and comprised 7% of the total fungal abundance after the bags were incubated in the field (Fig. 2). Moreover, in the mineral horizon, the abundance of this particular OTU in the mesh bags placed in the fertilized soils was 80% lower than those in the control soils (*p* < 0.05). Similarly, the abundance of the saprotrophic fungus *Myceliophthora heterothallica* was significantly higher in the fertilized soils of the mineral horizon (*p* < 0.05). The number of species in the mesh bags was similar in the control and fertilized plots at both horizons (Fig. S4).

When the total fungal communities, including all OTUs were compared, N fertilization did not result in any differences in the fungal community of the mesh bags in the organic horizon (R^2^ = 0.24, *p* = 0.3) or in the mineral horizon (R^2^ = 0.37, *p* = 0.1) (Fig. 3c, d).

### Chemical analysis of the organic matter in the mesh bags in the organic and mineral horizons

Mesh bags from the organic horizon showed similar amounts of C and N compared to the initial material, while a significant decrease for both C and N in the mineral horizon was observed (*p* < 0.05, Table 1). Input of carbon from outside the mesh bags (from C3 plant such as Norway spruce) that resulted in a decrease of ^13^C in the mesh bag content occurred at both horizons with respect to initial material. No significant changes in the isotopic composition (^13^C) or the pH of the mesh bags were found between control and fertilized treatments (Table 1). Estimation of the C input in the mesh bags ranged from 0.82 to 0.86 mg C g^−1^ sand, which corresponded to a 10% of the total C. However, there were no differences between the control and fertilized plots at both horizons. Nitrogen fertilization did not result in changes in the C and N content of the mesh bags, and the mesh bags placed in control plots showed similar values to those in the fertilized plots in both soil horizons. The C/N ratios of the mesh bags increased significantly in comparison to that of the initial material (on average 21% more with respect to the C/N ratio of the initial material). The C/N ratios tended to be higher in control plots than in fertilized ones, although this difference was not statistically significant. Since the ECM biomass in these forests has a C/N ratio of 20 (Wallander et al. 2003) and taking into account that new C in the mesh bags was 10%, the contribution of fungal biomass to the total increase of C/N was estimated to be 32%. Likewise, the initial amount of nitrates and ammonium in the mesh bags were 0.03 and 0.002 g kg^−1^, respectively. This corresponded to 3 and 0.2% of nitrates and ammonium with respect to total N, respectively. Thus, considering this inorganic N as initially leached out, their contribution to the total increase of C/N was estimated of 16%.

**Table 1:** Chemical characteristics and carbon decay constant rates of the mesh bag contents at the beginning of the experiment (initial material) and after 17 months of incubation in the humus and mineral layers of the control and fertilized plots at the Norway spruce forest

	Initial material^a^	Humus layer		Mineral layer	
		Control	Fertilized	Control	Fertilized
C (g kg^−1^)	12.1 (1.4) a	10.3 (0.4) a	10.0 (0.8) a	8.1 (0.4) b	8.6 (0.1) b
N (g kg^−1^)	1.02 (0.11) a	0.71 (0.05) a	0.70 (0.04) a	0.55 (0.02) b	0.62 (0.07) b
C/N	11.9 (0.1) a	14.7 (0.5) b	14.3 (0.3) b	14.8 (0.6) b	14.0 (0.1) b
∆13C (‰)	−13.36 (0.3) a	−15.6 (0.2) b	−15.1 (0.2) b	−15.0 (0.2) b	−15.2 (0.2) b
pH	ND	4.16 (0.02)	4.28 (0.03)	4.25 (0.09)	4.19 (0.03)
C decay constant rates (yr^−1^) ^b^		0.11 (0.02)	0.14 (0.04)	0.29 (0.03)	0.25 (0.06)

^a^Standard error is in parentheses (*n* = 3). Values followed by different lowercase letters indicate significant difference between treatments and the initial material (*p* < 0.05)

^b^Calculated assuming one-compartmental exponential model, C_final_ = C_initial_ * e^-kt^

**Fig. 4:**
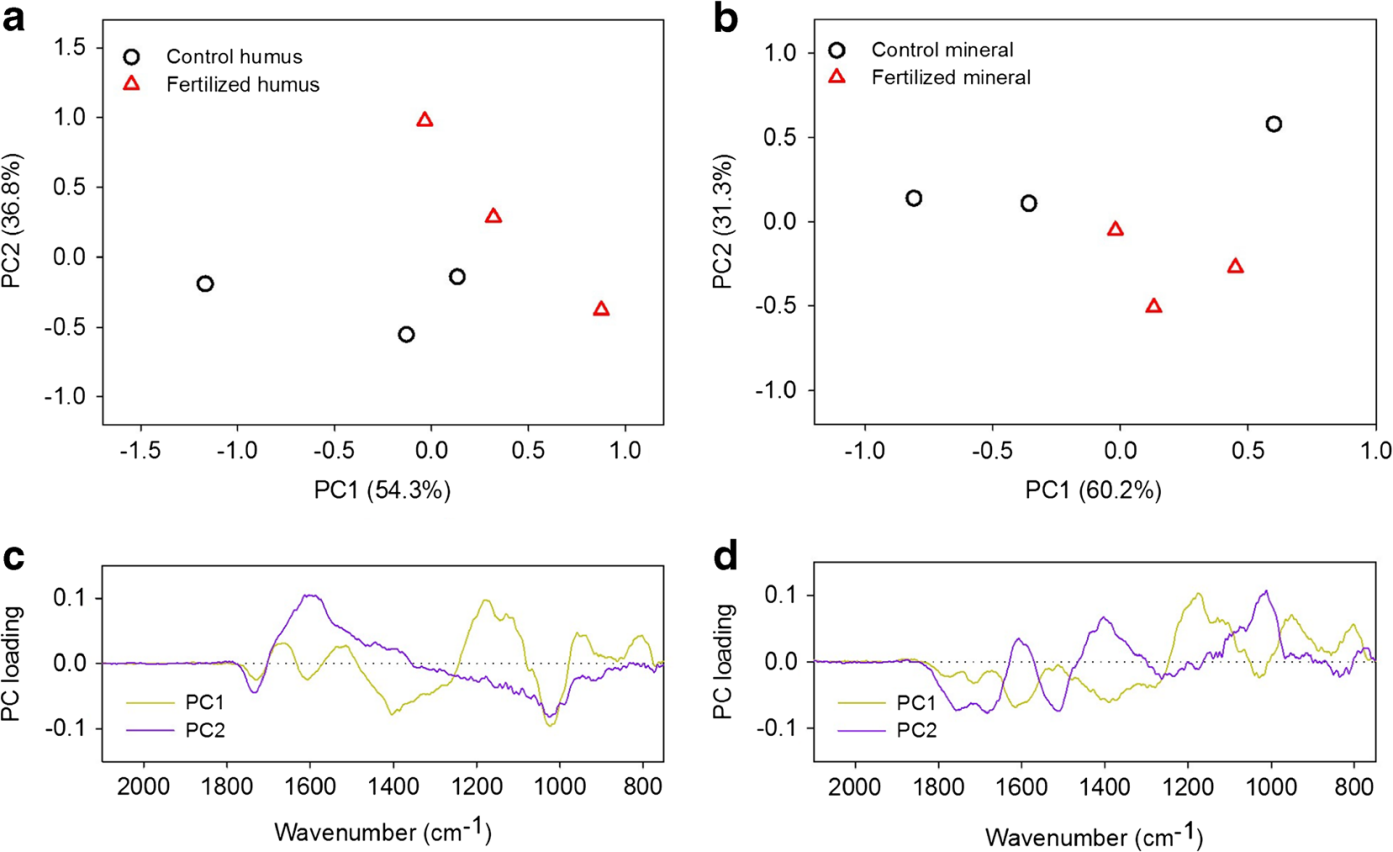
PCA scores (**a**, **b**) and loadings plot (**c**, **d**) of the infrared spectra of the dissolved organic matter obtained from the mesh bags after 17 months of incubation in the organic (on the left side) and mineral horizons (on the right side) of control and fertilized plots at the Norway spruce forest (*n* = 3)

Infrared spectra of the initial material were characterized by a dominant sharp peak, which is ascribed to nitrates and ammonium (peak at 1380 cm^−1^). This peak disappeared when the mesh bags were incubated in the field (Fig. S4). In addition, a peak in the initial material at 1170 cm^−1^ also disappeared at the end of the experiment. When compared the infrared spectra of mesh bags incubated in the field, PCA separated the organic matter in fertilized plots from the control plots in both horizons, with the differences in the mineral horizon being significant along principal component 2 (PC2) (Fig. 4b) (*p* < 0.05). PC2 accounted for 31.3% of the total variance and was mainly related to four vibrational regions: carbohydrate stretching, at 1100 cm^−1^; aliphatic deformation, at 1350–1450 cm^−1^; aromatic skeletal vibration, at 1510 cm^−1^; and carbonyl region, at 1620–1850 cm^−1^ (Fig. 4b). The PC2 loading plots revealed that this separation was based on higher content of carbohydrates in the control samples, while the fertilized ones seemed to be characterized by aromatic skeletal vibration and carbonyl groups (Fig. 4d).

**Fig. 5:**
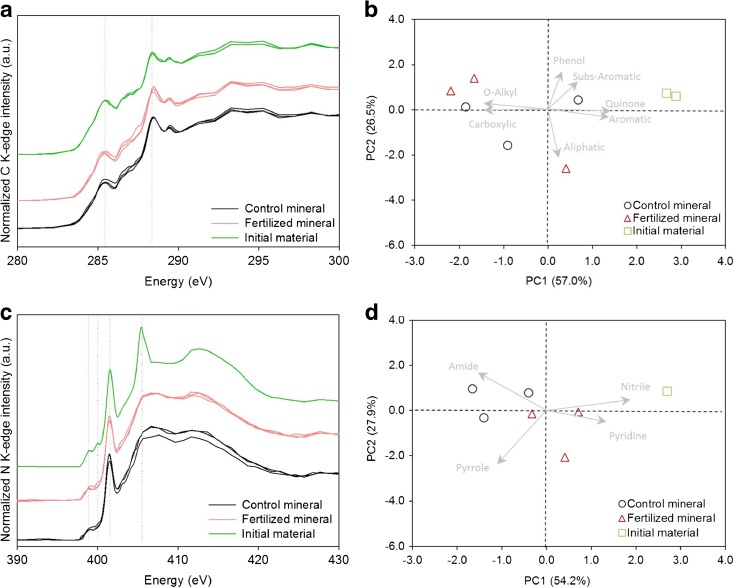
NEXAFS C1s (**a**) and N1s K-edge spectra of the organic matter (**c**), and the corresponding PCA scores and loading plot of their deconvoluted peak areas (**b**, **d**), obtained from the mesh bags at the beginning of the experiment (initial material) and after 17 months of incubation in the mineral horizon of the control and fertilized plots. Vertical dotted lines in the C1s K-edge spectra indicate aromatic-C, at 285.3 eV, and carboxyl-C, at 288.4 eV (*n* = 2 for the initial material, and *n* = 3 for the material inside the mesh bags). For N1s K-edge spectra, vertical dotted lines indicate heterocyclic-N (pyridine), at 398.8 eV; nitriles and aromatic-N, at 400 eV; amidic-N, at 401.4 eV; and nitrate-N, at 405.4 eV (*n* = 1 for the initial material, and *n* = 3 for the material inside the mesh bags). Spectra are pre-edge baseline corrected and post-edge normalized to 1. Loading factors were multiplied by 3 to improve visualization

As decomposition occurred more intensively in the mineral horizon (Table 1), the material of the mesh bags of this horizon was further investigated. Near edge X-ray absorption fine structure (NEXAFS) spectroscopy was applied to the samples of this horizon. The carbon K-edge spectra were similar for both control and fertilized treatments (Fig. 5a), as also indicated by the relative abundances estimated from the deconvoluted peaks (Table S1) and the fact that the treatments were grouped together in the PCA plot (Fig. 5b). However, weak trends were observed in the peak ascribed to carboxyl-C, which displayed higher intensity and relative abundance in the field samples as compared to the initial material, while the peak attributed to aromatic-C decreased (Fig. 5b, Table S1).

Regarding the N compounds, all samples showed predominance of amidic-N. The peaks at 398.8 eV and 400 eV, attributed to heterocyclic-N and nitrilic/heterocylic-N, were significantly lower in the incubated material in comparison to the initial material, with the control showing the lowest values (Fig. 5c, Table S1). PCA plots also revealed that field samples enriched in organic N such as amidic- and pyrrolic-N, while the abundance of heterocyclic-N (e.g. pyridine) and nitrilic-N diminished (Fig. 5d). A prominent peak at 405.4 eV, attributed to nitrate-N/ammonium-N, dominated the spectra of the initial material, but this was absent in the NEXAFS spectra of the mesh bags after 17 months (Fig. 5c).

## Discussion

### Fungal colonization and community composition of the mesh bags

Colonization of sand-filled mesh bags is usually dominated by ECM fungi, although a lesser amount of saprotrophic fungi is also present (Wallander et al. 2001, 2010). Wallander et al. (2010) found on average 70% ECM fungi in similar mesh bags without OM amendment in the same experimental site. However, the addition of compost in the present study resulted in a higher proportion of saprophytic fungi, and ECM fungi constituted 30–43% of the fungal abundance. Saprophytic fungi in the mesh bags were dominated by fungi originally present in the compost with 43% of the fungal reads, and only 12% corresponded to new colonizing saprotrophic fungi such as *Crystoporiopsis* spp. In contrast to our results, other studies have shown that the soil microbial community displaces the compost-borne microbial community of organic amendments applied to soil (Saison et al. 2006). It is likely that keeping the material confined in the mesh bags has prevented the displacement of the compost-borne community. Other researchers have used gamma-radiation to sterilize the OM added to the mesh bags and obtained community composition with ECM and saprotrophic fungal abundances of 70% and less than 9% relative to the total fungal abundance, respectively (Phillips et al. 2014). Nevertheless, it is important to bear in mind that the compost-borne fungi, which were present in mesh bags at both horizons and treatments, may or may not be adapted to the new conditions imposed in the environment. For instance, the saprotrophic fungi *Myceliophthora heterothallica* and *Aspergillus fumigatus* are compost-borne fungi that grow well under thermophilic conditions (Mehta and Satyanarayana 2013), but they would not be expected to grow at the low temperatures of the studied forest soils. In addition, some portion of these sequence reads likely arose from DNA extracted from dormant spores rather than active hyphae. Therefore, the high OTU abundances of some of these saprotrophic fungi may not be indicative of their decomposition activity.

Although a change in the fungal community structure at different N levels has been observed (Kjøller et al. 2012; Morrison et al. 2016), the fungal community, neither the total fungal community nor the ECM fungal community, did not change under N fertilization. It has been observed that the microbial community composition tends to be more significantly affected by mineral fertilization when the application is prolonged in time (Geisseler and Scow 2014), although there has also been found no effects on ECM fungal community composition if fertilization is balanced in nutrients (Hay et al. 2015). The similar fungal community composition between treatments in our 17-month experiment therefore points to the fact that more frequent fertilization and longer time studies are needed to obtain significant changes in the fungal community structure.

### Chemical changes in the organic matter of the mesh bags after fungal colonization

In partial agreement with our first hypothesis, the decomposition of OM in the mesh bags was, independently of the N treatment or soil horizon, accompanied by an increase of C/N ratio of the organic material. It seems that there was a demand for N, even in fertilized plots, and earlier studies have suggested that the Norway spruce forest in this experimental site is N limited (Bahr et al. 2015). The decrease in the organic C was likely due to the fungal and bacterial saprotrophic degradation, while the increase of the C/N ratio in the organic material could be due to the transfer of N to the plant by ECM fungi. A similar increase in C/N ratios was also observed in soil horizons from coniferous forests dominated by ECM fungi and was attributed to a preferential uptake and transfer of N by the ECM fungi (Lindahl et al. 2007). Nevertheless, the increase of the C/N ratio in our experiment was not exclusively due to N transfer by ECM fungi and almost half of this increase was due to the contribution of fungal biomass and inorganic N leaching in the mesh bags. It seems thereby that fungal colonization underwent with a concomitant action of saprotrophs and ectomycorrhizal fungi, which exploited the nutrient resources and modified the chemical composition of the OM supplied in the mesh bags. The role of bacteria in the decomposition of the organic matter inside the mesh bags cannot also be overseen, since they can constitute more than half of the microbial biomass in coniferous forest soils (Hartmann et al. 2012). Although the bacterial community composition is less affected by N addition (Freedman et al. 2015), bacteria may also have contributed to the decomposition of organic matter. However, it is unlikely that bacterial activity has contributed to the increase in C/N ratio of the OM since they cannot transport N over long distances.

Oxidative reactions are used by saprotrophic and ECM fungi in order to get access to nutrients in the OM (Martínez et al. 2011; Shah et al. 2016). These oxidative reactions include side chain oxidations, which can lead to an increase of carboxyl-C in the OM. The diminution of the abundance of substituted aromatic C and an increase of more oxidized compounds such as carboxyl-C in the mineral horizon may then indicate that oxidative mechanisms were likely occurring inside the mesh bags. There was also a stronger OM decomposition of the mesh bags in the mineral layer in comparison to the organic horizon, probably due to their different abiotic conditions. Environmental conditions such as moisture content affect the microbial activity and can be different between layers (Söderström 1979). For instance, the upper layer of the soil in these forests tends to dry out during summer, which could have resulted in lower activity leading to a lower decomposition rate in comparison to that of the mineral horizon.

### Changes in the organic N pool of the mesh bags after fungal colonization

Regarding our second hypothesis, there was a change in the proportion of organic N compounds in the mesh bags. The relative abundance of heterocyclic-N within the mesh bags decreased after fungal colonization, especially in the unfertilized plots. This modification in the relative abundance of organic N compounds was not mainly produced by the increase of fungal biomass in the mesh bags, since isotopic analysis and ergosterol measurements showed a similar fungal biomass between fertilized and unfertilized plots. There was then likely occurring a preferential use of the heterocyclic-N compounds by the fungi present in the mesh bags. It is known that fungi prefer taking up forms of N such as ammonium or glutamine if available, but fungi can also use, when such N forms are not available, other N sources such as nitrates, amines, amides, purines and pyrimidines (Marzluf 1997). Amidic-N is usually the dominant organic N source (up to 50% in many soils), but the fraction corresponding to heterocyclic-N, originated from nucleotide bases, chlorophyll or carbohydrates, can account up to 13–35% of the total organic N in the soil (Schulten and Schnitzer 1997; Talbot and Treseder 2010). Therefore, heterocyclic-N seems to be an important N source that fungi do not neglect under field conditions. Our results, however, do not exclude the fact that ECM fungi have simultaneously used other organic N sources such as amidic-N.

The larger ECM fungal abundance and lower heterocyclic-N relative abundance in the unfertilized plots compared to the fertilized plots, together with their trends of higher enrichment of C/N ratio, suggest a possible role of ECM fungi in utilizing and transporting the organic-N, especially the heterocyclic-N, to the host tree. Some ECM fungi have the capacity to use heterocyclic-N (Talbot and Treseder 2010), although there is still little known about its use by ECM fungi in forest soils. Among ECM fungi found in the mesh bags, the most abundant ectomycorrhizal fungal species, *Tylospora fibrillosa*, tended to decrease after N fertilization. *T. fibrillosa* has the capacity to use organic N sources such as the insoluble plant protein gliadin (Taylor et al. 2000), and has also been found to express manganese-dependent peroxidase activity, which could have given access to N sources associated to polyphenols (Chambers et al. 1999). Nevertheless, decomposition of OM by saprotrophic microorganisms (bacteria and fungi) might also have facilitated the access to these organic N forms for ECM fungi as suggested by other authors (Wu 2011).

## Conclusion

Fungal colonization of the organic material in the mesh bags resulted in chemical changes in the OM composition and was accompanied by an increase of C/N ratio of the mesh-bag content. The large abundance of ECM fungi in the mesh bags suggests that the depletion in N of OM during decomposition was due to the transfer of N by ECM fungi to the plant. Moreover, the use of novel spectroscopic techniques such as NEXAFS spectroscopy revealed that the relative abundance of heterocyclic-N diminished in the mesh bags from the mineral horizon of unfertilized plots in comparison to those of the initial material and fertilized ones. *T. fibrillosa* was the most abundant ECM species colonizing the mesh bags and may have contributed to the decline in heterocyclic-N. Further experiments under controlled conditions are needed to understand the importance of heterocyclic-N as N source for fungal community, especially for ECM fungi.

## Electronic supplementary material

Table S1 Relative abundance of carbon and nitrogen types in the mesh bag contents obtained from NEXAFS spectroscopy. The contents of the bags were analyzed at the beginning of the experiment (initial material) and after 17 months of incubation in the mineral layers of the control and fertilized plots at the Norway spruce forest. Values shown relative abundance of the deconvoluted peak area (mean ± standard error) for π* transitions of the C (a) and nitrogen K-edge NEXAFS spectra (b) with respect to the sum of the area of all π* transitions. (DOCX 17 kb)

Figure S1 Representative deconvolution of NEXAFS C1s (a) and N1s K-edge spectra of the organic matter (b) obtained from a sample. For C, deconvoluted peaks corresponded to quinone-C (284.3 eV), alkylated to carbonyl-substituted aromatic-C (285.3 eV), aromatic-C (286 eV), phenolic-C (286.6 eV), aliphatic-C (287.4 eV), carboxyl-C (288.4 eV), O-alkyl-C (289.3 eV), σ resonances (294.3 and 298.8 eV) and error function (289.9 eV). For N, deconvoluted peaks corresponded to the heterocyclic-N (398.8 eV), nitriles and aromatic-N (400 eV), amidic-N (401.4 eV), pyrrolic-N (402.7 eV), σ resonances (406 and 412.1 eV), and the error function step (403.2 eV) (DOCX 149 kb)

Figure S2 Species accumulation curves for all fungi (a) and for ectomycorrhizal fungi (b) for each sample. Curves were obtained by repeated random subsampling (DOCX 440 kb)

Figure S3 Ectomycorrhizal fungal OTUs and their corresponding relative abundance obtained from the mesh bags in the control and fertilized plots after 17 months of incubation (a) in the humusand (b) in the mineral layer (*n* = 3). Only OTUs with greater than 50 reads are shown (representing >99% of the total reads) (DOCX 330 kb)

Figure S4 Saprophytic fungal OTUs and their corresponding relative abundanceobtained from the mesh bags in the control and fertilized plots after 17 months of incubation (a) in the humusand (b) in the mineral layer (*n* = 3). Only OTUs with greater than 50 reads are shown (representing >99% of the total reads) (DOCX 500 kb)

Figure S5 Infrared spectra of the dissolved OM obtained from the mesh bags in control and fertilized plots. All spectra are normalized to the same total area over the wavenumber displayed. For each treatment, solid, dotted and dashed lines represent the spectrum for each replicate (DOCX 133 kb)
