# Supplementary material for: Chemical changes in organic matter after fungal colonization in a nitrogen fertilized and unfertilized Norway spruce forest
Source: Plant Soil. 2017 Jul 8;419(1):113–26. doi: 10.1007/s11104-017-3324-8 (PMC6959379; doi:10.1007/s11104-017-3324-8)
Supplement: Supplementary file 1 — Relative abundance of carbon and nitrogen types in the mesh bag contents obtained from NEXAFS spectroscopy. The contents of the bags were analyzed at the beginning of the experiment (initial material) and after 17 months of incubation in the mineral layers of the control and fertilized plots at the Norway spruce forest. Values shown relative abundance of the deconvoluted peak area (mean ± standard error) for π* transitions of the C (a) and nitrogen K-edge NEXAFS spectra (b) with respect to the sum of the area of all π* transitions. (DOCX 17 kb) [file 11104_2017_3324_MOESM1_ESM.docx]

Table S1.

| **Carbon type^a)^** | Energy (eV) | Initial material | Control | Fertilized | F-ratio | p-value |
| --- | --- | --- | --- | --- | --- | --- |
| Quinone-C | 284.3 | 5.6 (< 0.1) | 4.8 (0.2) | 4.5 (0.2) | 4.82 | 0.07 |
| Substituted aromatic-C | 285.3 | 12.9 (< 0.1) | 11.2 (< 0.1) | 11.7 (0.4) | 5.67 | 0.05 |
| Aromatic-C | 286.0 | 9.4 (0.1) | 8.6 (0.3) | 8.0 (0.4) | 2.90 | 0.14 |
| Phenolic-C | 286.6 | 5.3 (< 0.1) | 5.4 (< 0.1) | 5.2 (0.2) | 0.14 | 0.88 |
| Aliphatic-C | 287.4 | 19.1 (0.1) | 18.9 (< 0.1) | 19.4 (0.6) | 0.12 | 0.89 |
| Carboxyl-C | 288.4 | 22.7 (0.3) | 24.4 (0.2) | 24.6 (0.3) | 8.81 | 0.02 |
| O-alkyl-C | 289.3 | 25.2 (0.1) | 26.5 (0.4) | 26.8 (0.3) | 4.87 | 0.07 |
| **Nitrogen type^b)^** |  |  |  |  |  |  |
| Heterocyclic-N | 398.8 | 8.4 (-) | 8.0 (< 0.1) | 8.5 (0.2) | 8.61 | 0.04 |
| Nitrilic-N/heterocyclic-N | 400.0 | 12.4 (-) | 8.5 (0.3) | 9.1 (0.2) | 1.41 | 0.30 |
| Amidic-N | 401.4 | 54.2 (-) | 56.7 (0.3) | 55.1 (0.6) | 3.41 | 0.14 |
| Pyrrolic-N | 402.7 | 25.0 (-) | 26.9 (0.3) | 27.2 (0.5) | 0.29 | 0.62 |

^a)^ Statistical differences established between all treatments (initial material, control and fertilized). For the initial material, n = 2, and n = 3 for the material inside the mesh bags.

^b)^Statistical differences established between control and fertilized plots. For the initial material, n = 1, and n = 3 for the material inside the mesh bags.
