# Supplementary figures and images for: Chemical changes in organic matter after fungal colonization in a nitrogen fertilized and unfertilized Norway spruce forest
Source: Plant Soil. 2017 Jul 8;419(1):113–26. doi: 10.1007/s11104-017-3324-8 (PMC6959379; doi:10.1007/s11104-017-3324-8)

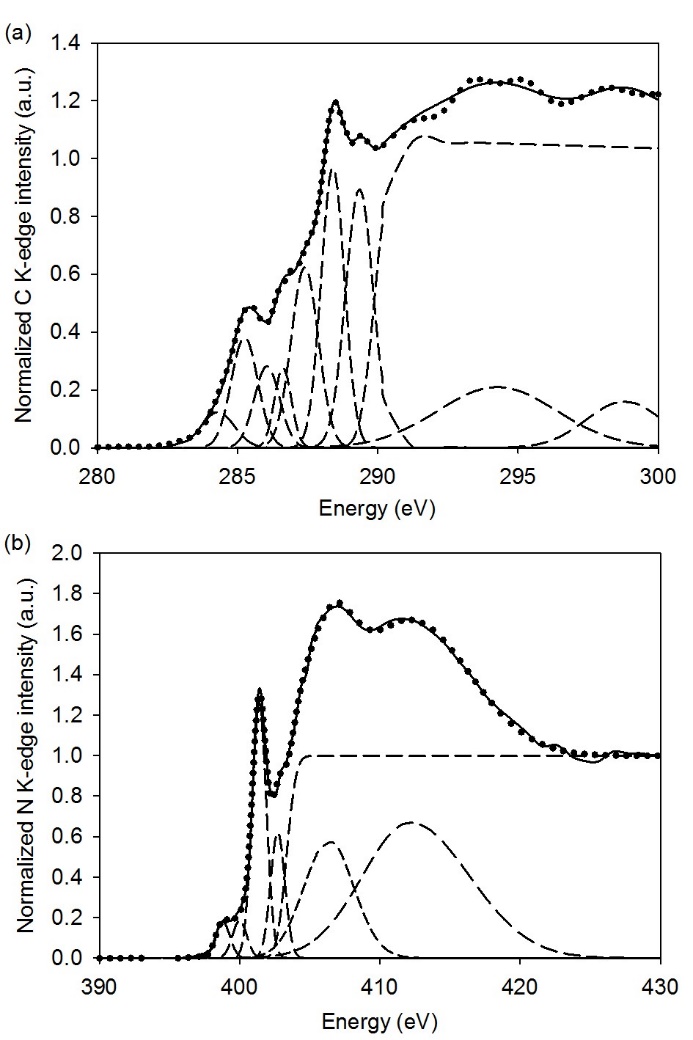


Fig. S1

Supplement: Supplementary file 2 — Representative deconvolution of NEXAFS C1s (a) and N1s K-edge spectra of the organic matter (b) obtained from a sample. For C, deconvoluted peaks corresponded to quinone-C (284.3 eV), alkylated to carbonyl-substituted aromatic-C (285.3 eV), aromatic-C (286 eV), phenolic-C (286.6 eV), aliphatic-C (287.4 eV), carboxyl-C (288.4 eV), O-alkyl-C (289.3 eV), σ resonances (294.3 and 298.8 eV) and error function (289.9 eV). For N, deconvoluted peaks corresponded to the heterocyclic-N (398.8 eV), nitriles and aromatic-N (400 eV), amidic-N (401.4 eV), pyrrolic-N (402.7 eV), σ resonances (406 and 412.1 eV), and the error function step (403.2 eV) (DOCX 149 kb) [file 11104_2017_3324_MOESM2_ESM.docx]

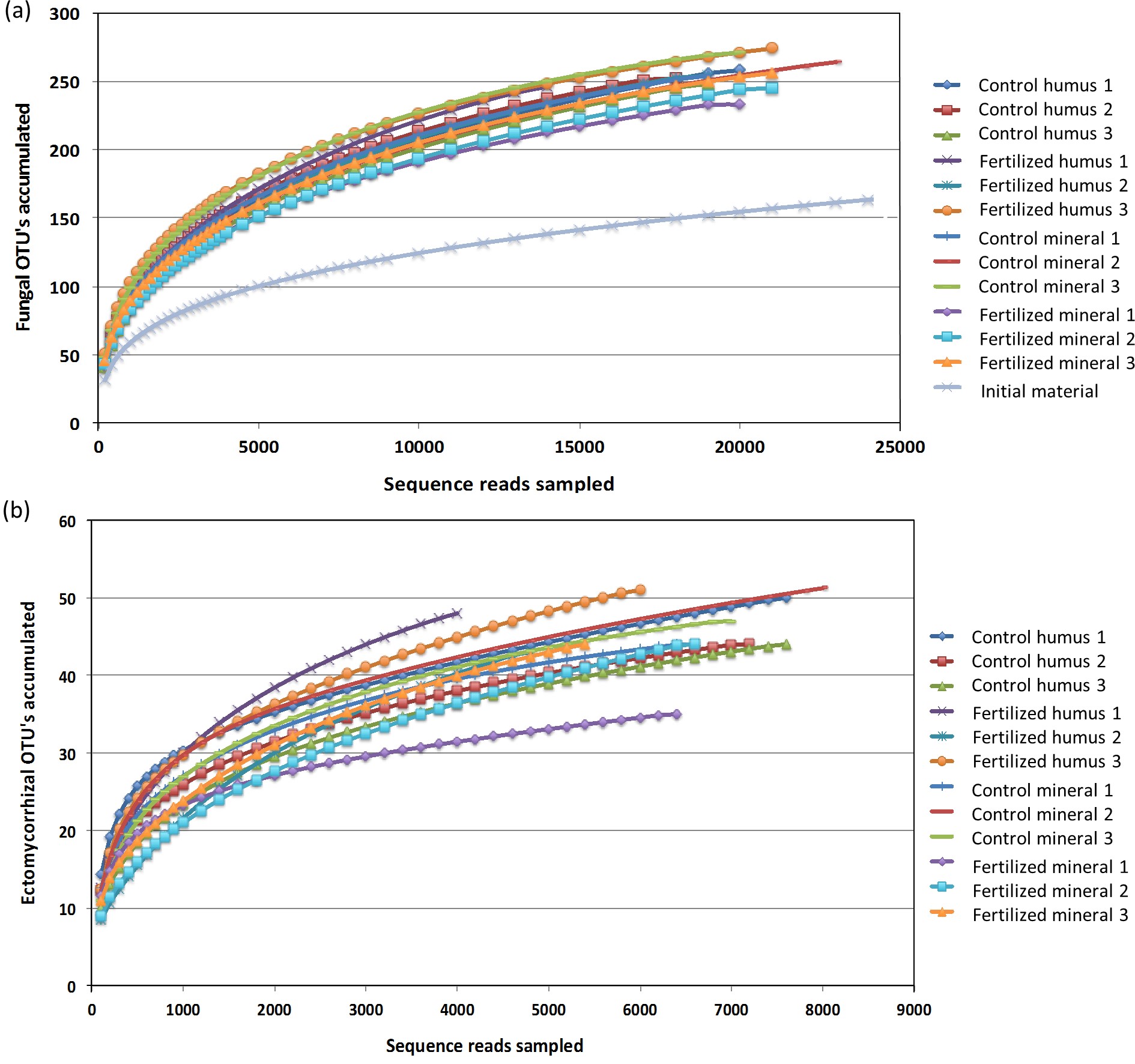


Fig. S2

Supplement: Supplementary file 3 — Species accumulation curves for all fungi (a) and for ectomycorrhizal fungi (b) for each sample. Curves were obtained by repeated random subsampling (DOCX 440 kb) [file 11104_2017_3324_MOESM3_ESM.docx]

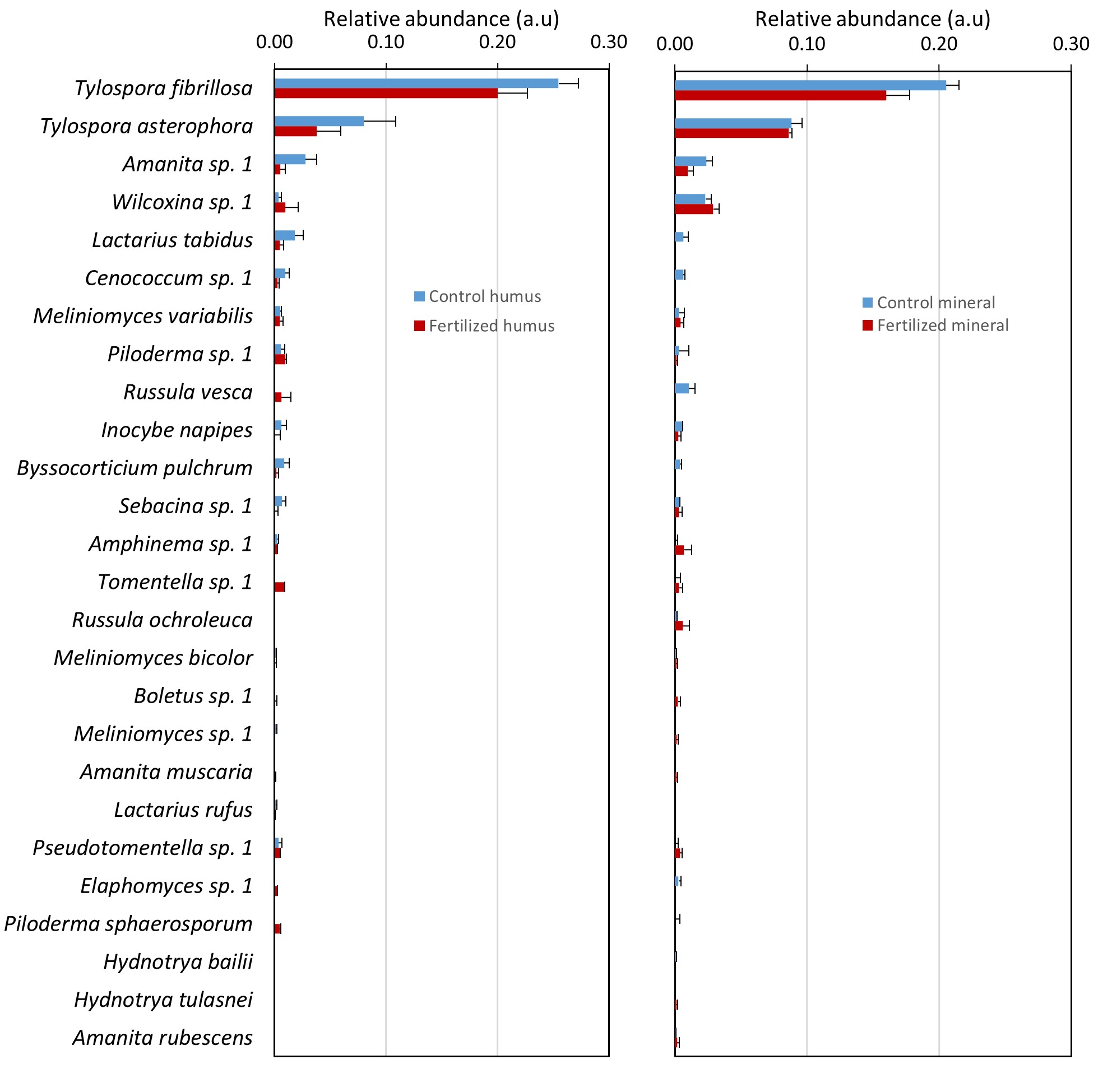


Fig. S3

Supplement: Supplementary file 4 — Ectomycorrhizal fungal OTUs and their corresponding relative abundance obtained from the mesh bags in the control and fertilized plots after 17 months of incubation (a) in the humusand (b) in the mineral layer (n = 3). Only OTUs with greater than 50 reads are shown (representing >99% of the total reads) (DOCX 330 kb) [file 11104_2017_3324_MOESM4_ESM.docx]

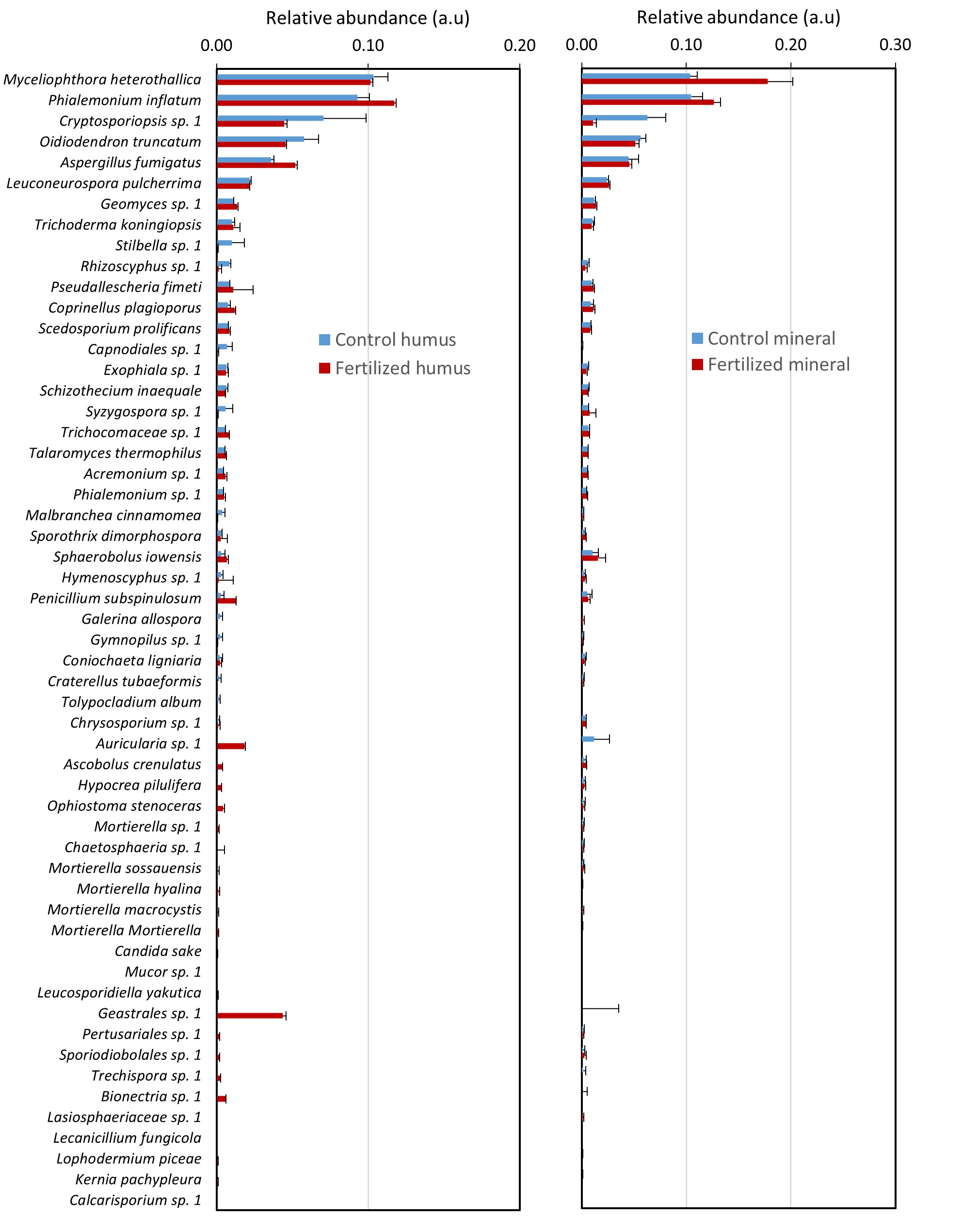


Fig. S4

Supplement: Supplementary file 5 — Saprophytic fungal OTUs and their corresponding relative abundanceobtained from the mesh bags in the control and fertilized plots after 17 months of incubation (a) in the humusand (b) in the mineral layer (n = 3). Only OTUs with greater than 50 reads are shown (representing >99% of the total reads) (DOCX 500 kb) [file 11104_2017_3324_MOESM5_ESM.docx]

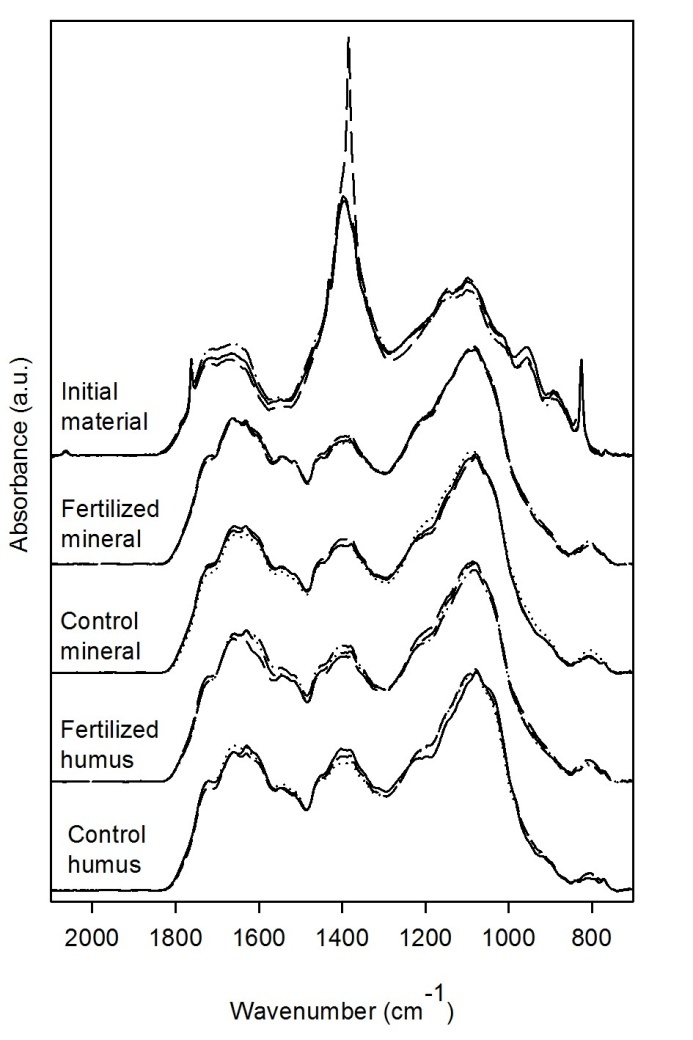


Fig. S5

Supplement: Supplementary file 6 — Infrared spectra of the dissolved OM obtained from the mesh bags in control and fertilized plots. All spectra are normalized to the same total area over the wavenumber displayed. For each treatment, solid, dotted and dashed lines represent the spectrum for each replicate (DOCX 133 kb) [file 11104_2017_3324_MOESM6_ESM.docx]
